# A chromosome-level reference genome for the common octopus, *Octopus vulgaris* (Cuvier, 1797)

**DOI:** 10.1093/g3journal/jkad220

**Published:** 2023-10-18

**Authors:** Dalila Destanović, Darrin T Schultz, Ruth Styfhals, Fernando Cruz, Jèssica Gómez-Garrido, Marta Gut, Ivo Gut, Graziano Fiorito, Oleg Simakov, Tyler S Alioto, Giovanna Ponte, Eve Seuntjens

**Affiliations:** Department of Neurosciences and Developmental Biology, University of Vienna, Vienna 1030, Austria; Department of Neurosciences and Developmental Biology, University of Vienna, Vienna 1030, Austria; Department of Biology, Lab of Developmental Neurobiology, Animal Physiology and Neurobiology Division, KU Leuven, Leuven 3000, Belgium; Department of Biology and Evolution of Marine Organisms, Stazione Zoologica Anton Dohrn, Naples 80121, Italy; Centro Nacional de Análisis Genómico (CNAG), Barcelona 08028, Spain; Centro Nacional de Análisis Genómico (CNAG), Barcelona 08028, Spain; Centro Nacional de Análisis Genómico (CNAG), Barcelona 08028, Spain; Centro Nacional de Análisis Genómico (CNAG), Barcelona 08028, Spain; Department of Biology and Evolution of Marine Organisms, Stazione Zoologica Anton Dohrn, Naples 80121, Italy; Department of Neurosciences and Developmental Biology, University of Vienna, Vienna 1030, Austria; Centro Nacional de Análisis Genómico (CNAG), Barcelona 08028, Spain; Department of Biology and Evolution of Marine Organisms, Stazione Zoologica Anton Dohrn, Naples 80121, Italy; Department of Biology, Lab of Developmental Neurobiology, Animal Physiology and Neurobiology Division, KU Leuven, Leuven 3000, Belgium; KU Leuven Institute for Single Cell Omics (LISCO), KU Leuven, Leuven 3000, Belgium; Leuven Brain Institute, KU Leuven, Leuven 3000, Belgium

**Keywords:** coleoid cephalopods, chromosome-scale, Hi-C, Octopus vulgaris

## Abstract

Cephalopods are emerging animal models and include iconic species for studying the link between genomic innovations and physiological and behavioral complexities. Coleoid cephalopods possess the largest nervous system among invertebrates, both for cell counts and brain-to-body ratio. *Octopus vulgaris* has been at the center of a long-standing tradition of research into diverse aspects of cephalopod biology, including behavioral and neural plasticity, learning and memory recall, regeneration, and sophisticated cognition. However, no chromosome-scale genome assembly was available for *O. vulgaris* to aid in functional studies. To fill this gap, we sequenced and assembled a chromosome-scale genome of the common octopus, *O. vulgaris*. The final assembly spans 2.8 billion basepairs, 99.34% of which are in 30 chromosome-scale scaffolds. Hi-C heatmaps support a karyotype of 1n = 30 chromosomes. Comparisons with other octopus species' genomes show a conserved octopus karyotype and a pattern of local genome rearrangements between species. This new chromosome-scale genome of *O. vulgaris* will further facilitate research in all aspects of cephalopod biology, including various forms of plasticity and the neural machinery underlying sophisticated cognition, as well as an understanding of cephalopod evolution.

## Introduction

Coleoid cephalopods (cuttlefish, squid, and octopus) comprise about 800 extant species characterized by highly diversified lifestyles, body plans, and adaptations. Cephalopod-specific traits, such as complex nervous systems (Young 1964; Hochner *et al.* 2006; Hochner 2012; Fiorito *et al.* 2014; Wang and Ragsdale 2019; Ponte *et al.* 2021), advanced learning abilities (reviewed in Marini *et al.* 2017), and the richness in body patterning considered to be involved in camouflaging and communication (Borrelli *et al.* 2006; Chiao and Hanlon 2019) have made this taxon ideal for studying evolutionary novelties. The neural plasticity of cephalopod brains and the existence of evidence for functionally analogous structures shared with mammalian brains have made cephalopods into a model comparative clade for neurophysiology research (Shigeno *et al.* 2018; Styfhals *et al.* 2022).

Despite the technical difficulties of sequencing their typically large and repetitive genomes, the available cephalopod genomes have given insights into the genomic basis for the evolution of novelty (Albertin *et al.* 2015, 2022; Kim *et al.* 2018; Li *et al.* 2020; Jiang *et al.* 2022; Marino *et al.* 2022; Schmidbaur *et al.* 2022). The first-published cephalopod genome, that of *Octopus bimaculoides* (Albertin *et al.* 2015), made it clear that cephalopod genomic novelties were not attributable to whole-genome duplication, as occurred in the vertebrate ancestor (Meyer and Schartl 1999; Dehal and Boore 2005). Comparisons of recently available chromosome-scale genome assemblies, including those of the Boston market squid *Doryteuthis pealeii* (Albertin *et al.* 2022) and the Hawaiian bobtail squid *Euprymna scolopes* (Schmidbaur *et al.* 2022), have shown the impact of genome reorganization on novel regulatory units in coleoid cephalopods. Still, it is not yet known how these units are made in terms of their gene content or their evolution in separate squid and octopus lineages. In this respect, it is crucial that the growing cephalopod genomics resources and approaches help obtain high-quality genomes for the established experimental species.

The common octopus, *Octopus vulgaris*, has long been used as a model for the study of learning and cognitive capabilities in invertebrates (reviewed in Young 1964; Marini *et al.* 2017), and is also used as a comparative system in the study of neural organization and evolution (Shigeno *et al.* 2018; Ponte *et al.* 2022). Furthermore, recent advances in the culture of this species' early life stages have increased its suitability for molecular approaches and have provided important developmental staging information (Deryckere *et al.* 2020).

One bottleneck to studying *O. vulgaris* is the lack of a chromosome-scale genome assembly. While the reported karyotype of *O. vulgaris* is 1n = 28 (Inaba 1959; Vitturi *et al.* 1982) or 1n = 30 (Gao and Natsukari 1990), to date there is no definitive answer. Existing genomic resources for *O. vulgaris* include a short read-based genome assembly (Zarrella *et al.* 2019), and a genome annotation based on the closely related *O. sinensis* genome (Li *et al.* 2020) that is supported with PacBio Iso-Seq reads and FLAM-seq curation (Styfhals *et al.* 2022; Zolotarov *et al.* 2022). These resources have been valuable in characterizing the molecular and cellular diversity of the developing brain (Styfhals *et al.* 2022), the evolution of cephalopod brains (Zolotarov *et al.* 2022), and the noncoding RNA repertoire unique to cephalopods (Petrosino *et al.* 2022). Further improvements to the *O. vulgaris* genome assembly and genome annotation will provide a valuable resource to the cephalopod and neuroscience communities.

Here we describe a chromosome-scale genome assembly and annotation of the common octopus, *O. vulgaris*. We have validated our assembly using available chromosome-scale genomes of octopus species (Li *et al.* 2020, Albertin *et al.* 2022; Jiang *et al.* 2022). Our analyses reveal large-scale chromosomal homologies, yet a pattern of local rearrangement within chromosomes between species.

## Materials and methods

### Sample collection

One adult male *Octopus vulgaris* (780 g body weight, specimen tube3-27.05.21-GP, BioSamples ERS14895525 and ERS14895526) was collected in the Gulf of Naples, Italy (40°48′04.1″N 14°12′32.7″E) by fishermen in May 2021. The animal was immediately sacrificed humanely following EU guidelines and protocols for the collection of tissues from wild animals (Andrews *et al.* 2013; Fiorito *et al.* 2015) (see Data Availability for animal welfare information). The central brain masses (optic lobes, OL; supra-esophageal mass, SEM; sub-esophageal mass, SUB) were dissected out (ERS14895525), and the spermatophores (ERS14895526) were collected as described in Zarrella *et al.* (2019). All dissections were carried out on a bed of ice in seawater, and the excised tissues were then weighed and flash-frozen in liquid nitrogen.

### High molecular weight genomic DNA extraction

High molecular weight genomic DNA (HMW gDNA) was extracted from a frozen spermatophore sample (160 mg) (ERS14895526) using a salt-extraction protocol at the Stazione Zoologica Anton Dohrn (Italy) following Albertin *et al.* (2022). Briefly, two cryopreserved sample aliquots were each lysed for 3 hours at 55°C in separate tubes of 3 mL lysis buffer containing proteinase K. Then 1 mL of NaCl (5 M) was added to each tube. The tubes were mixed by inversion and then spun down for 15 minutes at 10,000 rcf. The supernatants were then transferred to a new tube and 2 volumes of cold ethanol (100%) were added. The DNA precipitate was then spooled, washed, resuspended in elution buffer (10 mM Tris, 0.1 mM EDTA, pH 8.5), and stored at 4°C. The DNA concentration was quantified using a Qubit DNA BR Assay kit (Thermo Fisher Scientific), and the purity was evaluated using Nanodrop 2000 (Thermo Fisher Scientific) UV/Vis measurements.

### 10× genomics library preparation and sequencing

A 10 ng aliquot of the spermatophore HMW DNA was used to prepare a 10x Genomics Chromium library (Weisenfeld *et al.* 2017) at the National Center for Genomic Analysis (Centre Nacional d'Anàlisi Genòmica—CNAG, Spain) using the Chromium Controller instrument (10x Genomics) and Genome Reagent Kits v2 (10x Genomics) following the manufacturer's protocol. The library was indexed with both P5 and P7 indexing adaptors. The resulting sequencing library was checked that the insert size matched the protocol specifications on an Agilent 2100 BioAnalyzer with the DNA 7500 assay (Agilent).

The library was sequenced at CNAG with an Illumina NovaSeq 6000 with a read length of 2 × 151 bp, and was demultiplexed with dual indices (Supplementary Data 1).

### Long-read whole genome library preparation and sequencing

The spermatophore HMW DNA was also used to prepare one Oxford Nanopore Technologies (ONT) 1D sequencing library (kit SQK-LSK110) at CNAG. Briefly, 2.0 μg of the HMW DNA was treated with the NEBNext formalin-fixed paraffin-embedded DNA Repair Mix (NEB) and the NEBNext Ultra II End Repair/dA-Tailing Module (NEB). ONT sequencing adaptors were then ligated to the DNA, then the DNA was purified with 0.4 × AMPure XP Beads and eluted in Elution Buffer.

Two sequencing runs were performed at CNAG on an ONT PromethIon 24 using ONT R9.4.1 FLO-PRO 002 flow cells. The libraries were sequenced for 110 hours. The quality parameters of the sequencing runs were monitored by the MinKNOW platform version 21.05.8 (ONT) and base called with Guppy, version 5.0.11 (available through https://community.nanoporetech.com) (Supplementary Data 1).

### Omni-C library preparation and sequencing

A Dovetail Genomics Omni-C library was prepared at SciLifeLab (Solna, Sweden) using the flash-frozen brain tissue from the same individual used to generate the ONT long reads and 10x Genomics Chromium reads (ERS14895525). One hundred milligrams of brain tissue were pulverized to a fine powder using a mortar and pestle under liquid nitrogen. Two 20 mg aliquots of the pulverized tissue were fixed in PBS with formaldehyde and disuccinimidyl glutarate (DSG) and were prepared according to the manufacturer's protocol as two separate libraries. To increase the final complexity, the two libraries bound to streptavidin beads were pooled together into a single tube before P7 indexing PCR. The amplified library was sequenced at SciLifeLab on an Illumina NovaSeq 6000 with a read length of 2 × 150 bp, and was demultiplexed with one index (Supplementary Data 1).

### Nuclear genome assembly

Sequencing produced 77 Gb of ONT whole genome sequencing (WGS) reads (27.5 × coverage) and 230.25 Gb of 10x Genomics linked reads (77.7 × coverage). These data were assembled with the CNAG Snakemake assembly pipeline v1.0 (https://github.com/cnag-aat/assembly_pipeline) to obtain an optimal base assembly for further Hi-C scaffolding. In brief, this pipeline first preprocessed the 10x reads with *LongRanger basic* v2.2.2 (https://github.com/10XGenomics/longranger) and filtered the ONT reads with *FiltLong* v0.2.0 (https://github.com/rrwick/Filtlong), and then the ONT reads were assembled with both *Flye* v2.9 (Kolmogorov *et al.* 2019) and *NextDenovo* v2.4.0 (Hu *et al.* 2023). The following evaluations were run on both assemblies and after each subsequent step of the pipeline: Benchmarking Universal Single-Copy Orthologs (BUSCO) v5.2.2 (Manni *et al.* 2021) with *metazoan_odb10* and *Merqury* v1.1 (Rhie *et al.* 2020) to estimate the consensus accuracy (QV) and k-mer statistics, and *fasta-stats.py* for contiguity statistics. The best contig assembly was obtained with *NextDenovo* (see assembly metrics Supplementary Data 2), so the remaining steps of the pipeline were run on this assembly (Supplementary Fig. 1 and Data 2).

The assembly was polished with 10x Illumina and ONT reads using *Hypo* v1.0.3 (Kundu *et al.* 2019); collapsed with *purge_dups* v1.2.5 (Guan *et al.* 2020); and then scaffolded with the 10x Chromium reads using *Tigmint* v1.2.4 (Jackman *et al.* 2018), *ARKS* v1.2.2 (Coombe *et al.* 2018) and *LINKS* v1.8.6 (Warren *et al.* 2015) following the Faircloth's Lab protocol (http://protocols.faircloth-lab.org/en/latest/protocols-computer/assembly/assembly-scaffolding-with-arks-and-links.html). The specific parameters and versions used to assemble the *O. vulgaris* specimen are listed in Supplementary Data 3. Finally, 310 scaffolds shorter than 1 Kb were removed from the assembly. This assembly was used for scaffolding with Omni-C data.

### Omni-C scaffolding

The Omni-C reads (863.85 million read pairs) were then mapped to the assembly (Supplementary Data 4) using the recommended procedure from Dovetail Genomics (https://omni-c.readthedocs.io/en/latest/fastq_to_bam.html). In short, the reads were mapped to the reference using *bwa mem* v0.7.17-r1188 (Li 2013) with flags *-5SP -T0*, converted to a sorted .*bam* file, and filtered to reads with a minimum mapping quality of 30 with *samtools* v1.9 (Li *et al.* 2009) with *htslib* v1.9, and filtered to keep uniquely mapping pairs with *pairtools* v0.3.0 (Open2C *et al.* 2023). The minimum mapping quality threshold of 30 was used to accommodate for the organism's heterozygosity and repetitiveness (1.22 and 68.68%, respectively, see Supplementary Data 5). After excluding PCR duplicates and improperly mated reads with *pairtools*, 231.59 million Hi-C read pairs were used to scaffold the assembly with *YaHS* v1.1 (Zhou *et al.* 2023) in the default mode, thus initially detecting and correcting errors in contigs, introducing breaks at misjoins.

### Generation of the Hi-C heatmaps and manual curation

We then manually curated the scaffolded assembly using an editable Hi-C heatmap to improve the assembly's quality and to correct misassemblies. The process described below was repeated for five rounds until there were no obvious improvements to make based on the Hi-C heatmap signal.

*Chromap* v0.2.3 (Zhang *et al.* 2021) was used to align the Omni-C reads to the genome with a read alignment quality cutoff of Q0. The resulting *.pairs* file (quality cutoffs: 2,10) was converted using *awk* v 4.2.1(Aho *et al.* 1988) to a *.longp* file, a format used by *Juicebox Assembly Tools* (Dudchenko *et al.* 2018). We ran the script *run-assembly-visualizer.sh* from the *3D-DNA* pipeline (Dudchenko *et al.* 2017) on the *.longp* file to generate a *.hic* file. The *generate-assembly-file-from-fasta.awk* script from the *3D-DNA* pipeline (Dudchenko *et al.* 2017), and the *assembly-from-fasta.py* from the *Artisanal* pipeline (Bredeson *et al.* 2022) were used to generate the *.assembly* files necessary to curate the *.hic* heatmap file in *Juicebox Assembly Tools* (Dudchenko *et al.* 2018).

The resulting *.hic* heatmap file was visualized using the visualization tool *Juicebox* v1.11.08 (Durand *et al.* 2016). Using the signal in the Hi-C heatmap we corrected the order and orientation of contigs within the chromosome-scale scaffolds, and placed small contigs and scaffolds onto the chromosome-scale scaffolds. A new *.fasta* assembly was generated from the corrected *.assembly* file by using the *assembly-to-fasta.py* script from the *Artisanal* pipeline.

The corrected assembly was aligned to the chromosome-scale *O. sinensis* (GCA_006345805.1) (Li *et al.* 2020), *O. bimaculoides* (GCA_001194135.2) (Albertin *et al.* 2022), and *A. fangsiao* (Jiang *et al.* 2022) genomes using *minimap2* v2.24 (Li 2018), *snakemake* v7.19.1-3.11.1 (Köster and Rahmann 2012) and the *snakemake* script *GAP_dgenies_prep* (https://doi.org/10.5281/zenodo.7826771). The resulting *.paf* file was visualized with *D-GENIES* v1.4.0 (Cabanettes and Klopp 2018). Regions of the *O. vulgaris* chromosome-scale scaffolds that had ambiguous Hi-C heatmap signal, or regions that had no obvious homology to other *Octopus* spp. chromosome-scale scaffolds were removed from the chromosome-scale scaffolds and retained as smaller scaffolds at the end of the genome assembly *.fasta* file. Scaffolds were renamed based on homology with *O. bimaculoides* chromosomes.

### Decontamination

After curation, we ran the *BlobToolKit* INSDC pipeline (Challis *et al.* 2020), using the NCBI *nt* database (updated in December 2022) and the following BUSCO *odb10* databases: eukaryota, fungi, bacteria, metazoa, and mollusca. This analysis identified 226 scaffolds either matching the phylum Mollusca or having no-hit in the database (Supplementary Fig. 2). A total of 47 small scaffolds matching other phyla (Supplementary Data 6 and Fig. 3) were considered contaminants and removed from the assembly. This scaffolded and decontaminated assembly was then carried forward for annotation and comparative analyses, and is available at https://denovo.cnag.cat/octopus and the INSDC (The European Nucleotide Archive [ENA], NCBI, and The DNA Data Bank of Japan [DDBJ]) accession number GCA_951406725.1.

### Nuclear genome annotation

The gene annotation of the octopus genome assembly was obtained by combining transcript alignments, protein alignments, and *ab initio* gene predictions as described below. A flowchart of the annotation process is shown in Supplementary Fig. 4.

Repeats present in the genome assembly were annotated with *RepeatMasker* v4-1-2 (Smit *et al.* 2013–2015) using the custom repeat library available for Mollusca. Moreover, a new repeat library specific to the assembly was made with *RepeatModeler* v1.0.11. After excluding repeats from the resulting library that were part of repetitive protein families by performing a *basic local alignment search tool* (*BLAST*) (Altschul *et al.* 1990) search against *Uniprot*, *RepeatMasker* was rerun with this new library to annotate species-specific repeats.

PacBio Iso-Seq reads from several developmental stages were downloaded from NCBI (PRJNA718058, PRJNA791920, and PRJNA547720) (García-Fernández *et al.* 2019; Deryckere *et al.* 2021; Zolotarov *et al.* 2022). Bulk RNA-seq from an adult octopus (Petrosino *et al.* 2022) was downloaded from the *ArrayExpress* database under accession number E-MTAB-3957. The short and long reads were aligned to the genome using *STAR* v-2.7.2a (Dobin *et al.* 2013) and *minimap2* v2.14 (Li 2018) with the option *-x splice:hq*. Transcript models were subsequently generated using *Stringtie* v2.1.4 (Pertea *et al.* 2015) on each .*bam* file, and then all the transcript models were combined using *TACO* v0.6.3 (Niknafs *et al.* 2017). High-quality junctions to be used during the annotation process were obtained by running *Portcullis* v1.2.0 (Mapleson *et al.* 2018) after mapping with *STAR* and *minimap2*. Finally, *PASA* assemblies were produced with *PASA* v2.4.1 (Haas *et al.* 2008). The *TransDecoder* program, part of the *PASA* package, was run on the *PASA* assemblies to detect coding regions in the transcripts.

The complete proteomes of *O. vulgaris*, *O. bimaculoides,* and *Sepia pharaonis* were downloaded from *UniProt* in October 2022 and aligned to the genome using *Spaln* v2.4.03 (Iwata and Gotoh 2012).

*Ab initio* gene predictions were performed on the repeat-masked assembly with 2 different programs: *Augustus* v3.3.4 (Stanke *et al.* 2006) and *Genemark-ES* v2.3e (Lomsadze *et al.* 2014) with and without incorporating evidence from the RNA-seq data. Before gene prediction, *Augustus* was trained with octopus-specific evidence. The gene candidates used as evidence for training *Augustus* were obtained after selecting *Transdecoder* annotations that were considered complete and did not overlap repeats, clustering them into genes, and selecting only one isoform per gene. These candidates were aligned to the *Swissprot* NCBI database with *blastp* v2.7.1 (Altschul *et al.* 1990) to select only those with homology to proteins. The final list of candidate genes was made of 1,764 genes with *BLAST* hits to known proteins with e-values smaller than 10^−9^ and greater than 55% identity.

Finally, all the data were combined into consensus coding sequence models using *EVidenceModeler* v1.1.1 (EVM) (Haas *et al.* 2008). Additionally, UTRs and alternative splicing forms were annotated via two rounds of *PASA* annotation updates. Functional annotation was performed on the annotated proteins with *Blast2go* v1.3.3 (Conesa *et al.* 2005). First, a *DIAMOND* v2.0.9 *blastp* (Buchfink *et al.* 2021) search was made against the *nr* database. Furthermore, *Interproscan* v5.21-60.0 (Jones *et al.* 2014) was run to detect protein domains on the annotated proteins. All these data were combined by *Blast2go* v1.3.3, which produced the final functional annotation results.

Identification of long noncoding RNAs (lncRNAs) was done by first filtering the set of *PASA*-assemblies that had not been included in the annotation of protein-coding genes to retain those longer than 200 bp and not covered more than 80% by repeats. The resulting transcripts were clustered into genes using shared splice sites or significant sequence overlap as criteria for designation as the same gene.

### Nuclear genome and annotation completeness assessment

The final *O. vulgaris* genome assembly, the annotated transcripts, the proteins from the annotated transcripts, and the other available octopus genomes were assessed for completeness using BUSCO databases as described above (Materials and Methods—Genome Assembly). To compare the qualities of each assembly, we used *fasta_stats* (Chapman *et al.* 2011) shown in (Table 1). We calculated the percentage of bases in the chromosome-scale scaffolds (Table 1) with *bioawk* v1.0 (https://github.com/lh3/bioawk).

**Table 1.** Octopus genome assembly statistics.

Assembly	Number of scaffolds	Number of contigs	Scaffold sequence total	Scaffold N50/L50	Number of scaffolds >50 KB	% of the bases in chromosome-scale scaffolds
Final chromosome-scale *O. vulgaris* genome	226	2758	2800.4 MB	118.9 MB/9	57	99.34
Pre-curation scaffolded assembly *O. vulgaris*	768	2776	2801.6 MB	118.3 MB/9	296	95.82
Chromosome-scale *O. bimaculoides* (Albertin *et al.* 2022)	145,326	713,915	2342.5 MB	96.9 MB/9	85	95.46
Chromosome-scale *O. sinensis* (Li *et al.* 2020)	13,516	20,491	2719.2 MB	105.9 MB/10	1800	86.09
Chromosome-scale *A. fangsiao* (Jiang *et al.* 2022)	6409	9099	4341.1 MB	169.7 MB/10	1769	93.05

### Mitogenome assembly and annotation

To assemble the mitochondrial genome we employed a strategy that uses a reference bait to select the mitochondrial nanopore reads, assembles those reads into a single circular contig, and then performs two rounds of polishing. To obtain the mitochondrial sequences, all ONT reads with a mean quality of ≥10 were mapped with *minimap2* v2.24 (Li 2018) against the circular complete, 15,744 bp mitochondrial genome of another specimen of *O. vulgaris* (NC_006353.1) (Yokobori 2004) with the minimap2 parameter *-ax map-ont*. We retained all reads with a mapping quality ≥13. Approximately 5,000 ONT reads passed these filters including 15 reads accounting for 181,644 total basepairs (12 × coverage) with a mean length of 12,112 bp.

All the retained ONT reads were assembled with *Flye* v2.9 (Kolmogorov *et al.* 2019) using the options *flye –scaffold -i 2 -g 15744 –nano-raw –min-overlap 7000*. This produced one circular contig. The -*i 2* option specified for *flye* caused this contig to be polished twice with the input ONT reads. After polishing the length of the circular contig was 15,651 bp, and a web *blastn* search revealed that it spanned the length of the NC_006353.1 mitochondrial genome. The circular mitogenome contig was rotated and oriented as follows. First, we annotated the contig using *MITOS* v2.1.3 (Bernt *et al.* 2013) with parameters *-c 5 –linear –best -r refseq81m*. Second, we used the coordinates in the *results. bed* file to orient the mitogenome, so it starts with the conventional tRNA Phenyl-Alanine (trnF) (Formenti *et al.* 2021).

To evaluate the assembly accuracy, we aligned the selected ONT reads back to the assembly with *minimap2* and visually inspected the alignment using *IGV* v2.14.1 (Robinson *et al.* 2023). Finally, the xcOctVulg1 mitogenome was aligned against the mitogenome of other species using *DNAdiff* v1.3 from *mummer* package v3.23 (Kurtz *et al.* 2004). These species included the mitogenomes of another specimen of *O. vulgaris* (NC_006353.1), *O. sinensis* (NC_052881.1), *O. bimaculoides* (NC_029723.1), and *A. fangsiao* (AB240156.1). From these pairwise alignments, we calculated the percent identity.

## Results and discussion

### DNA sequencing

Sequencing the ONT WGS library yielded 8.3 million ONT PromethIon reads containing 82.57 billion base pairs (Gbp) with 29.47 × coverage per library. Sequencing of the 10x Genomics Chromium library yielded 762 million read pairs containing 228.69 Gbp with 81.64 × coverage per library. The Omni-C library sequencing yielded 863.85 million read pairs, containing 259.16 Gbp of data with 33.02 × coverage. Details about sequence data can be found in Supplementary Data 1.

### Manual curation and decontamination of the assembly

**Fig. 1.**
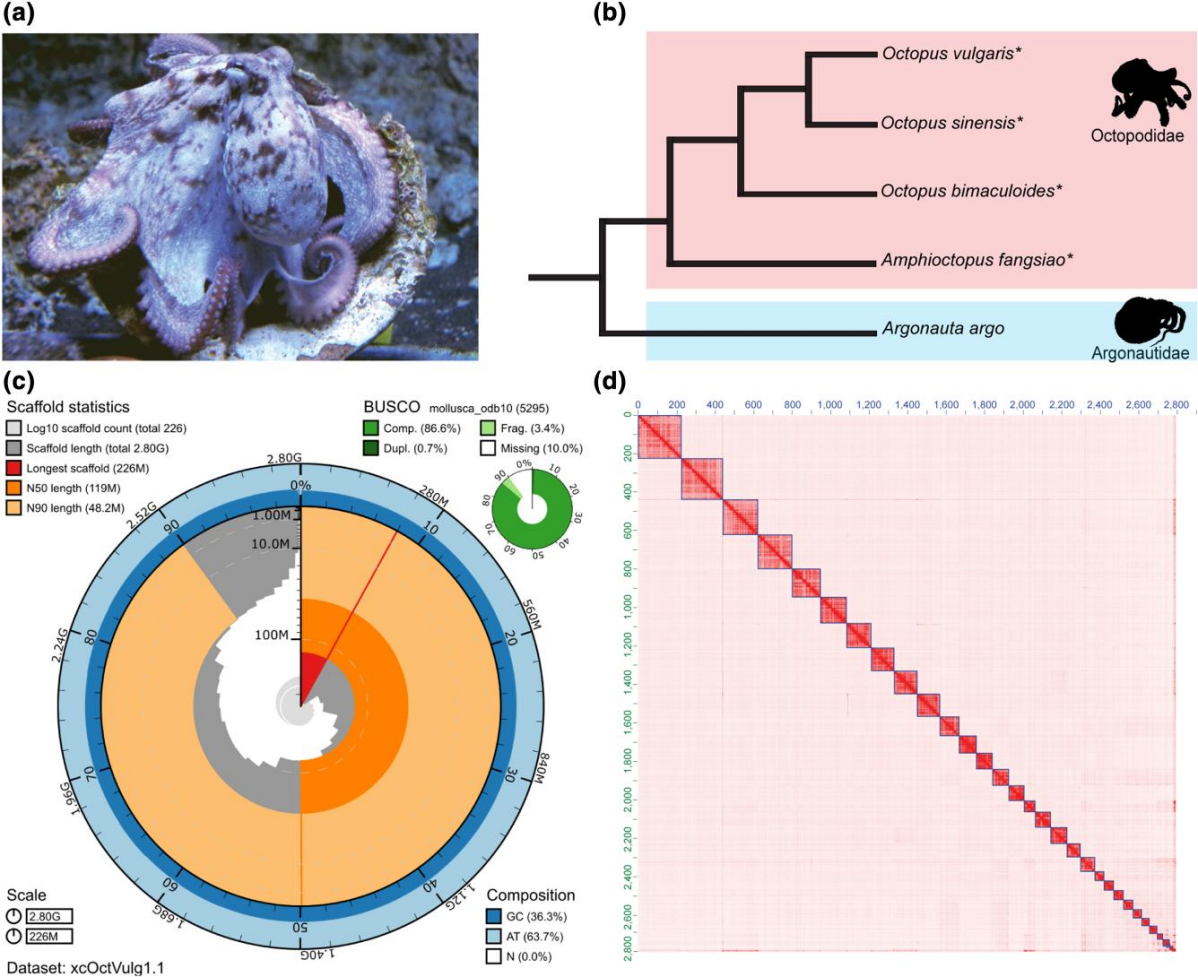
*Octopus vulgaris* assembly statistics and quality control. a) A specimen of *O. vulgaris*. b) A cladogram showing the phylogenetic relationship between the compared species and the family Argonautidae as an outgroup (Taite *et al.* 2023). Chromosome-scale genome assemblies are available for the starred species (*). c) The snail plot generated using Blobtools2 (Challis *et al.* 2020) shows that the final version of the chromosome-scale *O. vulgaris* assembly has N50 of 119 Mb, the longest scaffold is 225 Mb long, and a BUSCO score for complete genes of 86.6% against the *mollusca_odb10* database. d) The Hi-C heatmap of the final genome assembly shows 30 chromosome-scale scaffolds with very few sequences in unplaced scaffolds. Photography credit: panel a - © Antonio, Valerio Cirillo (BEOM SZN).

Manually curating the genome assembly improved the quality of the final assembly, as 495 scaffolds were placed in the chromosome-scale scaffolds, and 47 additional scaffolds were removed through the contamination analysis (Table 1). The final 2.80 Gb assembly, xcOctVulg1.1, has a scaffold N50 of 118.9 Mb, an N90 of 18.2 Mb, QV39 and gene completeness estimated using BUSCO v5.3.2 with *mollusca_odb10* of C:86.5% (S:85.8%, D:0.7%), F:3.4%, M:10.1%, n:5295 (Fig. 1c). The BUSCO score with *metazoa_odb10* for the final assembly is C:92.3% (S:91.8%, D:0.5%), F:2.7%, M:5.0% (Table 2). The statistics for all intermediate assemblies are shown in Supplementary Data 2. Also, in Supplementary Fig. 3 we show that the final assembly has been properly decontaminated.

**Table 2.** *Metazoa_odb10* BUSCO scores for availble octopus genomes.

Genome	Complete BUSCO	Single BUSCO	Duplicated BUSCO	Fragmented BUSCO	Missing BUSCO
Chromosome-scale *O. vulgaris*	92.3% [881]	91.8% [876]	0.5% [5]	2.7% [25]	5.0% [48]
Contig-level *O. vulgaris* (Zarrella *et al.* 2019)	63.1% [602]	62.6% [597]	0.5% [5]	24.8% [237]	12.1% [115]
Chromosome-scale *O. bimaculoides* (Albertin *et al.* 2022)	94.6% [903]	94.2% [899]	0.4% [4]	3.2% [31]	2.2% [20]
Chromosome-scale *O. sinensis* (Li *et al.* 2020)	95.7% [913]	90.5% [863]	5.2% [50]	2.6% [25]	1.7% [16]
Chromosome-scale *A. fangsiao* (Jiang *et al.* 2022)	93.5% [892]	91.6% [874]	1.9% [18]	3.5% [33]	3.0% [29]

### The octopus karyotype

The genome assembly from this study contains 30 large scaffolds with Hi-C heatmap signal that is consistent with each scaffold representing a single chromosome (Fig. 1d) and resembles the Hi-C heatmaps of other chromosome-scale octopus genome assemblies (Li *et al.* 2020; Albertin *et al.* 2022; Jiang *et al.* 2022). The first reported *O. vulgaris* karyotypes from Japan and Italy were 1n = 28 chromosomes (Inaba 1959; Vitturi *et al.* 1982), but later studies also using *O. vulgaris* individuals sampled in Japan reported at 1n = 30 (Gao and Natsukari 1990). The karyotype 1n = 30 have been reported in four other octopus species: *Callistoctopus minor, Amphioctopus fangsiao, Cistopus sinensis*, and *Amphioctopus areolatus* (Gao and Natsukari 1990; Adachi *et al.* 2014; Wang and Zheng 2017). The only exception is *Hapalochlaena maculosa* which does not have a confirmed karyotype, but 47 linkage groups were suggested for this species (Whitelaw *et al.* 2022).

**Fig. 2.**
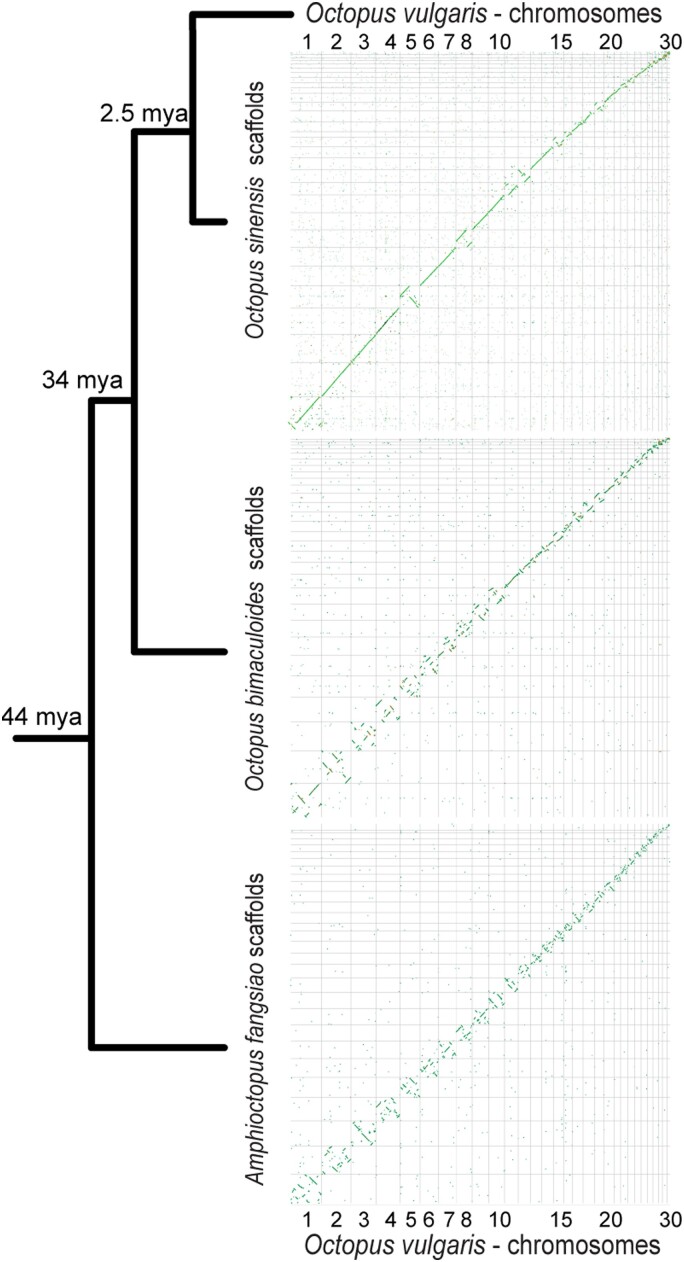
Comparative analyses of available chromosome-scale Octopodidae genomes. The figure shows the inferred phylogenetic relationship (Amor *et al.* 2017; Jiang *et al.* 2022; Taite *et al.* 2023) and the inferred divergence times (Amor *et al.* 2019; Jiang *et al.* 2022) of four octopus species. The diagrams show genome-genome alignments for each species compared to *O. vulgaris*.

In light of the recent taxonomic designation of a new species *O. sinensis* (East Asian Common Octopus) from the previously synonymous *O. vulgaris* (Gleadall 2016; Amor *et al.* 2017, 2019; Amor 2023), this suggests that the reported *O. vulgaris* karyotypes probably belong to *O. sinensis*. Dot plot analyses, described below, show that *O. vulgaris* and *O. sinensis* share 30 homologous, largely collinear, chromosomes (Fig. 2).

The final version of the *O. vulgaris* genome was aligned to the genomes of three octopus species, *O. sinensis*, *O. bimaculoides*, and *A. fangsiao* (Fig. 2). *O. vulgaris* and *O. sinensis* have few inversions between homologous, collinear chromosomes. General chromosomal collinearity was also observed in comparison to *O. bimaculoides* (Fig. 2). We found large-scale inversions (megabase-scaled, larger than 1Mb) throughout the genomes of two species. The overall sequence similarity is lower compared to the previous pair, and a greater number of chromosomal rearrangements are present. This is expected considering that *O. bimaculoides* and the *O. vulgaris*-*O. sinensis clade* diverged around 34 million years ago (mya) (Jiang *et al.* 2022), while *O. sinensis* and *O. vulgaris* diverged just 2.5 mya (Amor *et al.* 2019). In Fig. 2, the collinearity between *O. vulgaris* and *A. fangsiao* chromosomes is visible only in chromosomes 3 and 20. Furthermore, as *A. fangsiao* is the most distant to *O. vulgaris* of the compared species, the genomes are even more rearranged.

Our whole-genome alignment analyses support the hypothesis that *O. vulgaris, O. sinensis*, *O. bimaculoides,* and *A. fangsiao* share 30 homologous chromosomes (Fig. 2). Given the divergence time of these species, these results suggest that the karyotype of the common ancestor of this clade, and perhaps the common ancestor of octopuses, also had 30 chromosomes that still exist in extant species.

Karyotype stability was described in the squid lineage (Decapodiformes) on loliginid and sepiolid squids (Albertin *et al.* 2022). This study has suggested that the smaller karyotype found in octopuses (1n = 30) compared to squids (1n = 46) results from secondary fusions of a more ancestral squid chromosomal complement. Recently, it has been suggested that chromosomal fusions impact recombination, as well as chromosomal nuclear occupancy, in mice (Vara *et al.* 2021). Therefore, chromosomal fusions in the common ancestor of the octopus lineage might be one of the drivers of diversification, as they change chromosomal interactions and are hypothesized to lead to the formation of novel regulatory units (Vara *et al.* 2021). Such events are important in light of understanding the emergence of octopus-specific traits. We infer from the genome-genome comparisons that a similar pattern of intrachromosomal rearrangements with the conservation of individual chromosomes is seen in octopus species, as described in squids (Albertin *et al.* 2022). However, the loliginids and sepiolids are estimated to have diverged 100 mya (Albertin *et al.* 2022), while the genera *Octopus* and *Amphioctopus* are estimated to have diverged 44 mya (Jiang *et al.* 2022). Therefore, a more-distant species' chromosome-scale genome is needed to claim karyotype stasis in Octopodiformes. Nevertheless, future comparative studies of the genomes of these closely related species will shed light on the evolutionary history of octopuses as a separate lineage of coleoid cephalopods. In addition to this, *O. vulgaris* is a model animal in neurobiological studies, and having a high-quality genome will facilitate further studies of the cephalopod brain.

### Nuclear genome annotation

In total, we annotated 23,423 protein-coding genes that produce 31,799 transcripts (1.36 transcripts per gene) and encode 30,121 unique protein products. We were able to assign functional labels to 53.5% of the annotated proteins. The annotated transcripts contain 8.42 exons on average, with 87% of them being multi-exonic (Table 3). In addition, 1,849 long noncoding transcripts have been annotated. The number of protein-coding genes annotated here is slightly lower than those reported for other octopus genome assemblies, like *O. sinensis* (Li *et al.* 2020). After checking the general statistics of both annotations (Table 3), we observed that the genes annotated here tended to be longer (both in the number of exons and global length). After comparing both methods, we believe that the main cause of the difference in observed gene lengths is the source of the transcriptomic data, as the inclusion of long-read Iso-Seq data in the annotation process is known to result in less fragmented and longer annotations.

**Table 3.** Genome annotation statistics.

	OctVul6B annotation	Osinensis ASM634580v1 (Li *et al.* 2020)
Number of protein-coding genes	23,423	31,676
Median gene length (bp)	20,288	4,403
Number of transcripts	31,799	31,676
Number of exons	168,570	184,658
Number of coding exons	161,430	180,943
Median UTR length (bp)	1,255	441
Median intron length (bp)	2,467	1,520
Exons/transcript	8.42	5.83
Transcripts/gene	1.36	1
Multi-exonic transcripts	87%	81%
Gene density (gene/Mb)	8.36	11.7

### Nuclear genome and annotation completeness assessment

The BUSCO score was calculated for the *O. vulgaris*, *O. bimaculoides*, *O. sinensis*, and *A. fangsiao* genomes. For the chromosome-scale *O. vulgaris* genome, the BUSCO score for a whole-genome nucleotide sequence using the metazoan reference dataset was 92.3% for complete genes (954 core genes). The full score is in Table 2. This is an improvement considering the BUSCO score of the previous *O. vulgaris* genome assembly (GCA_003957725.1) for complete genes was 63.1% (Zarrella *et al.* 2019). Additionally, we assessed the completeness of the annotated proteome and transcriptome by calculating the BUSCO score against the *metazoa_odb10* and *mollusca_odb10* databases (Supplementary Data 2).

### Mitogenome assembly and annotation

The mitogenome assembly of the *O. vulgaris* specimen (xcOctVulg1) has a length of 15,651 bp and contains 13 protein-coding, 23 ncRNA, 2 rRNA, and 21 tRNA genes. The ONT read alignment to the mitogenome shows high consensus support for each nucleotide except for 16 positions (Supplementary Fig. 5). These 16 positions are single nucleotide polymorphisms, not indels, and the base at each position is the base with the highest coverage in the reads at that position (Supplementary Fig. 6). Therefore, the mitochondrial genome has a high per-base accuracy.

The percentages of identity (see Supplementary Data 7) between the *O. vulgaris* and other octopus mitochondrial genome sequences are consistent with the phylogeny topology (Fig. 2, Supplementary Data 7), and previous research on octopus taxonomy. The mitochondrial genome of the specimen collected in Japan and identified as *O. vulgaris* (NC_006353.1) shows a higher identity to *O. sinensis* (99.85%) than to our *O. vulgaris* specimen (96.79%). The 3.21% difference between the mitogenomes of the specimen from this study and NC_006353.1 is close to the estimated divergence rate (∼2% divergence/million years (Arbogast and Slowinski 1998)) for *O. vulgaris* and *O. sinensis* [estimated time of divergence: 2.5mya (Amor *et al.* 2019)]. These results suggest that the specimen collected in Japan and identified as *O. vulgaris* (NC_006353.1) is more likely to be *O. sinensis*. This possibility is consistent with recent morphological, molecular, and geographic delimitations made between the *O. sinensis* and *O. vulgaris* species complex (Gleadall 2016; Amor *et al.* 2017, 2019; Amor 2023).

## Conclusion

*Octopus vulgaris* is an important emerging model in comparative neuroscience, cognition research, and evolutionary studies of cephalopods. The chromosome-scale genome assembly and annotation reported here provide an improved reference for single-cell multi-omics and the study of noncoding regions and gene regulatory networks, which require the context of chromosome-scale sequences. This assembly and annotation will also facilitate many avenues of cephalopod research, in particular analyses of genome evolutionary trends in octopus and cephalopods compared to other invertebrates. Furthermore, the chromosome-scale *O. vulgaris* genome assembly will allow the estimation of chromosome rearrangement rates, the emergence of novel coding and noncoding genes among octopuses, and the turnover rates of putative regulatory regions. The scientific interest in *O. vulgaris* as a model animal in many fields including (evolutionary) developmental biology and neuroscience will be facilitated by the availability of a high-quality genome.

These efforts may help bridge the traditional *O. vulgaris* research on neurobiology, behavior, and development to the molecular determinants involved in these fields.

## Data Availability

The data are available at https://denovo.cnag.cat/octopus. On the International Nucleotide Sequence Database Collaboration databases (ENA, NCBI, DDBJ) the genome is available at accession GCA_951406725.1, and the data in BioProject PRJEB61268. Euthanizing cephalopods solely for tissue removal does not require authorization from the National Competent Authority under Directive 2010/63/EU and its transposition into National Legislation. Samples were taken from local fishermen, and humane killing followed principles detailed in Annex IV of Directive 2010/63/EU as described in the Guidelines on the Care and Welfare of Cephalopods (Fiorito *et al.* 2015). The sampling of octopuses from the wild included in this study was authorized by the Animal Welfare Body of Stazione Zoologica Anton Dohrn (Ethical Clearance: case 06/2020/ec AWB-SZN). Genomes of *O. sinensis* (GCA_006345805.1) (Li *et al.* 2020) and *O. bimaculoides* (GCA_001194135.2) (Albertin *et al.* 2022) were downloaded from NCBI, while the *A. fangsiao* genome (Jiang *et al.* 2022) was downloaded from Figshare (https://figshare.com/s/fa09f5dadcd966f020f3). The supplementary material generated in this study was deposited on figshare (https://doi.org/10.25387/g3.24119760).
